# A diagnostic medical physicist’s guide to the American College of Radiology Fluoroscopy Dose Index Registry

**DOI:** 10.1002/acm2.13227

**Published:** 2021-03-25

**Authors:** A. Kyle Jones, Kevin A. Wunderle, Dustin A. Gress, Michael Simanowith, Kay Zacharias‐Andrews, Shalmali Dharmadhikari, Xinhui Duan, Don‐Soo Kim, Usman Mahmood, Steve D. Mann, Jeffrey M. Moirano, Rebecca A. Neill, Alan H. Schoenfeld

**Affiliations:** ^1^ The University of Texas MD Anderson Cancer Center Houston TX USA; ^2^ Cleveland Clinic Cleveland OH USA; ^3^ American College of Radiology Reston VA USA; ^4^ Emory University Atlanta GA USA; ^5^ The University of Texas Southwestern Medical Center Dallas TX USA; ^6^ Boston Children’s Hospital Boston MA USA; ^7^ Memorial Sloan Kettering Cancer Center New York NY USA; ^8^ Duke University Medical Center Durham NC USA; ^9^ University of Washington Seattle WA USA; ^10^ Montefiore Medical Center New York NY USA

## 1 INTRODUCTION

The American College of Radiology (ACR) computed tomography (CT) Dose Index Registry (DIR) has been extraordinarily successful, with dose indices collected for over 102 million CT examinations to date.^1^, ^2^ The CT DIR has provided an ongoing source of normative clinical data which has been the gold standard for national and international benchmarking.^3^

The largest existing normative dataset for fluoroscopically guided procedures is the Radiation Doses in Interventional Radiology (RAD‐IR) study, with a data collection period covering the mid‐ to late 1990’s.^4^, ^5^, ^6^ The RAD‐IR study included 2142 clinical interventional fluoroscopy procedures, performed at one of seven sites using a single fluoroscope make and model. Much has changed since the late 1990's, including the scope and number of fluoroscopically guided procedures performed; fluoroscope technology, including the introduction of flat panel image receptors and variable added filtration; informatics, including widespread implementation and availability of the Digital Imaging and Communications in Medicine (DICOM) Radiation Dose Structured Report (RDSR); and regulation, including mandatory reporting of reference air kerma (K_a,r_) in the United States starting in 2006.^7^

More recently, reporting of fluoroscopy dose indices has been included as a Merit Based Incentive Payments System (MIPS) quality measure,^8^ and the ACR National Radiology Data Registry (NRDR^TM^) has been recognized as a Qualified Clinical Data Registry (QCDR) for MIPS participation. Finally, fluoroscopy is used in a very wide range of medical procedures, ranging from diagnostic procedures such as barium swallow, barium enema, and cystography to complex image‐guided interventions such as aortic aneurysm repair and hepatic embolization.

The American College of Radiology Fluoroscopy Dose Index Registry (DIR) is the latest addition to the NRDR^TM^, and the first modality to join Computed Tomography (CT) in the DIR. Launching the DIR is a challenging process, requiring expertise in radiology, fluoroscopy, diagnostic medical physics, and informatics to configure the necessary hardware and software, map clinical facility procedure names to a lexicon of more than 200 standard procedure names, and use summary data reports to perform dose audits and evaluate the clinical use of fluoroscopy. The diagnostic medical physicist, with wide‐ranging content expertise in imaging physics, informatics, and medical imaging equipment, can lend their expertise and guidance to the healthcare enterprise and their healthcare institution during this process.

The purpose of this review is to provide guidance for sites wishing to participate in the Fluoroscopy DIR, and specifically to provide guidance for the diagnostic medical physicist who is assisting a healthcare organization in implementing the ACR Fluoroscopy DIR. The mechanics of participation will be discussed, as will common pitfalls in the process. This review is based largely on the experiences of the diagnostic medical physicists at the nine sites that participated in the pilot phase of the Fluoroscopy DIR. The pilot phase involved data collection for interventional radiology procedures from March 1, 2018 through December 31, 2019, and manual collection of data regarding fluoroscope configuration and use as well as the accuracy and stability of fluoroscope‐reported dose indices.^9^ Two checklists to aid in preparation for a site launch of the Fluoroscopy DIR have been provided at the end of this review. After reading this review, a site should be able to assemble a Fluoroscopy DIR team and launch the DIR while avoiding some of the major pitfalls that may otherwise be encountered along the way. We recommend that readers of this review also bookmark the NRDR^TM^ Support Page for quick reference when needed.

## 2 STEP 1. ASSEMBLE YOUR TEAM

A wide array of expertise is required to ensure a successful launch of the ACR Fluoroscopy DIR at your institution. Key team members and their roles are described briefly in this section.

### 2.1 NRDR^TM^ user profiles

Healthcare organizations that have registered for the NRDR^TM^ have a corporate account within the NRDR^TM^. One or more user profiles can be assigned to any user account associated with a corporate account. These profiles should be reviewed to determine which, if any, of the Fluoroscopy DIR team members should be assigned specific roles within the site corporate account. Among the NRDR^TM^ user profiles, three specific administrative roles are defined, ^10^ which are described in further detail below.

#### 2.1.1 Corporate account administrator (CAA)

The CAA is the top level administrative role defined in the NRDR^TM^ for a corporate entity (note that a corporate entity may be comprised of any number of facilities). Every participating corporate entity must identify one CAA when they enroll in the NRDR^TM^. The CAA assigns all other administrative roles for the site corporate account. A CAA can perform most of the functions of other users, with the exception of viewing detailed facility data. The CAA does not need to be the director or manager at the facility but serves as the point of contact for all NRDR^TM^ issues for their associated facilities.

#### 2.1.2 Facility administrator (FA)

The FA is the top level administrative role for an individual facility in the NRDR^TM^ and has the most comprehensive permissions for a single facility, including managing other user accounts. Only one FA may be active for a facility at any time. Every participating site must identify an FA when they add a facility to a NRDR^TM^ Corporate account. The FA can manage accounts and data access across all registries for that facility and can view detailed facility data. A single user may have the FA role at multiple facilities.

#### 2.1.3 Registry administrator (RA)

The third administrative role, RAs are authorized by FAs to manage Facility User accounts. They control access to a specific registry, and may perform all functions of a Facility User.

#### 2.1.4 Service user

Service Users are authorized by CAAs to create and edit scanner mappings in the TRIAD® Site Server configuration and to perform exam name mapping at the corporate account level. However, a Service User cannot view data or reports, and therefore some team members may need both the Service User and Facility User profiles assigned to their user account.

#### 2.1.5 Facility user

A Facility User can perform exam name mapping at the facility level and view reports.

### 2.2 Fluoroscopy DIR team roles

These are not official administrative roles or user profiles in the DIR, however, identifying the right personnel to play the following roles is key to a successful implementation of the Fluoroscopy DIR.

#### 2.2.1 Physician champion

The physician champion is the coordinator and director of the implementation process, and is essential to securing and maintaining institutional support for implementation of the ACR Fluoroscopy DIR. Sites in states with requirements for fluoroscopy radiation protocol committees or similar committees may find their physician champion on one of these committees. At other sites, there may be a physician who is interested and engaged in quality improvement efforts who would make a great champion. One place to start looking is in your interventional radiology department, as interventional radiologists have much of the domain knowledge that will be required during later phases of the implementation process.

#### 2.2.2 Diagnostic medical physicist

The qualified diagnostic medical physicist is instrumental to a successful implementation of the Fluoroscopy DIR, as their domain knowledge should span the technical aspects of fluoroscopy, radiation dosimetry, informatics, and basic knowledge of the clinical aspects of fluoroscopy. Owing to their broad knowledge domain and scope of practice,^11^ diagnostic medical physicists can also facilitate effective communication among the other key team members.

#### 2.2.3 Information technology (IT) specialist

Implementing the Fluoroscopy DIR requires interfacing with multiple hospital systems and servers, requiring the expertise of an IT specialist, particularly one who is familiar with imaging informatics.

#### 2.2.4 TRIAD® site server administrator

The TRIAD® Site Server Administrator is responsible for ongoing maintenance of the TRIAD® Site Server. Both this role and the role of IT Specialist may be played by the same individual.

#### 2.2.5 Lead fluoroscopy technologist

Later stages of implementation will require the expertise of team members who have a deep understanding of the clinical aspects of fluoroscopy, particularly as it relates to the mapping of site‐specific facility clinical procedure names to the ACR Common lexicon. While the physician champion may possess this expertise, especially an interventional radiologist, they may not have the time necessary to devote to the task of procedure mapping. It is also possible that some diagnostic medical physicists may possess the necessary domain knowledge to assist in the procedure mapping process.

## 3 STEP 2. COMPLETE THE APPLICATION PROCESS AND CREATE YOUR NRDR^TM^ ACCOUNT

Sites wishing to participate in any registry that is part of the NRDR^TM^, including the ACR Fluoroscopy DIR, must complete the application process and create corporate and facility accounts in the NRDR^TM^.^12^ The step‐by‐step guide on the ACR website walks a facility through the process of getting started with the NRDR^TM^. If a site already has an NRDR^TM^ account, they only need to complete an addendum to their existing participation agreement to participate in the Fluoroscopy DIR. No additional registration or fees are required for a facility currently participating in the CT DIR or General Radiology Improvement Database (GRID) registries.

## 4 STEP 3. DOWNLOAD AND INSTALL THE TRIAD® SITE SERVER SOFTWARE

The TRIAD® Site Server software^13^ allows a corporate account to collect, de‐identify, and transmit data securely from their facilities to the NRDR^TM^ registries. The TRIAD® Site Server software must be downloaded, installed, configured, and maintained by a site administrator on a dedicated physical or virtual server behind the site’s firewall.

A single TRIAD® Site Server can support an entire corporate account, including multiple facilities and participation in multiple NRDR^TM^ registries.

## 5 STEP 4. CONNECTING FLUOROSCOPES TO THE TRIAD® SITE SERVER

DICOM RDSR from fluoroscopes at participating facilities must be transferred to the TRIAD® Site Server, where they are anonymized before they are forwarded to the ACR DIR Central Server. Fluoroscopes can be connected to the TRIAD® Site Server in a number of different configurations. While the ACR prefers that data be sent directly from fluoroscopes to the local TRIAD® Site Server, other configurations are not prohibited, and may be more efficient for sites with many fluoroscopes or multiple facilities. Only fluoroscopes that produce a DICOM RDSR are eligible to be connected to the registry, secondary capture dose sheets are not accepted and will not be processed.

Configuring fluoroscopes to create and transfer RDSR to another DICOM node may require service level access. This step is typically performed by the local service engineer under the guidance of the diagnostic medical physicist.

### 5.1 Direct connection to the TRIAD® site server

The simplest configuration is to establish a direct DICOM connection between each fluoroscope and the local TRIAD® Site Server, and then configure each fluoroscope to automatically create and transfer the RDSR to the Site Server after each procedure. Prior to configuring the fluoroscope, the local TRIAD Site Server must be installed and properly configured. Three pieces of information will be needed from the TRIAD® Site Server administrator to configure each fluoroscope:

1. The host name and/or Internet Protocol (IP) address of the TRIAD® Site Server.
2. The Application Entity Title (AET) of the TRIAD® Site Server.
3. The port number for the DICOM Storage SCP service on the TRIAD® Site Server.

### 5.2 Transfer to the TRIAD® site server through a radiation dose index monitoring (RDIM) system

Diagnostic medical physicists are already familiar with RDIM systems,^14^ and facilities that already have their fluoroscopes connected to an RDIM may elect to have the RDIM automatically forward RDSR to the TRIAD® Site Server. It is very important if choosing this configuration that the RDIM forward the RDSR without modification. The default behavior of some RDIM systems when exporting RDSR is to “rebuild” the RDSR using information parsed and stored from the original RDSR. In this scenario, fields may be missing, and the Device Observer Name (typically the Station Name) of the creating fluoroscope may be replaced with that of the RDIM, preventing the RDSR from being associated with the correct fluoroscope. Facilities electing this type of configuration are encouraged to contact their RDIM vendor early in the enrollment process to ensure proper configuration and avoid these potential pitfalls.

### 5.3 Auto routing to the TRIAD® site server from the facility picture archiving and communication system (PACS)

Facilities with the necessary in‐house PACS administrator expertise may elect to configure their PACS to automatically forward all RDSR from specific modalities or Station Names to the TRIAD® Site Server. This simplifies fluoroscope DICOM configuration as only a single DICOM destination is needed on each fluoroscope. Further, this destination likely already exists and is used to archive images. It is also possible for the PACS system to forward only to the TRIAD® Site Server or in parallel to the TRIAD® Site Server and an RDIM using this architecture.

### 5.4 Configuring fluoroscopes on the TRIAD® site server

Any fluoroscope from which the facility wishes to send RDSR to the DIR Central Server must be configured in the TRIAD® Site Server configuration console using the Station Name of the fluoroscope. The Station Name is part of the DICOM metadata (0008,1010), and while it is not a mandatory tag, most clinical imaging equipment populates this tag. The facility is responsible for ensuring that the Station Name has been configured using a unique identifier for all fluoroscopy systems that send RDSR to the DIR. RDSR from systems that are not mapped will be cached on the TRIAD® Site Server for 14 days, after which they will be deleted. Facilities can filter out specific fluoroscopes from sending data to the registry by *not* configuring these systems in the TRIAD® Site Server configuration console.

It is recommended that the Station Name be the *only* configuration information entered for a fluoroscope, as *all* fluoroscope configuration information must match for RDSR to be processed by the TRIAD® Site Server.

## 6 STEP 5. DATA VALIDATION

There are numerous failure modes that can interrupt data transfer between a facility and the DIR Central Server. Data validation is a quality assurance process that seeks to minimize interruptions in data transfer, minimize data bias, and maximize data fidelity. Regular data validation minimizes missing time periods, fluoroscopes, or procedure types in the data submitted from facilities to the DIR Central Server. Diagnostic medical physicists are experts in quality assurance and quality control, and can assist in designing appropriate quality assurance processes for data validation.

### 6.1 Initial data validation

Initial data validation focuses on ensuring that data are being received and processed at the ACR DIR Central Server from all fluoroscopes that a facility intends to connect to the Fluoroscopy DIR. The facility should verify that the full anonymized RDSR, with the Station Name of the creating fluoroscope, is being received from each connected fluoroscope. The “Fluoro/DR Summary of Data Submitted,” “Fluoro Dose Information By Exam,” and “Fluoro Exam Detail” reports, which are available in the DIR Portal, can be used to perform initial data validation.

### 6.2 Periodic data validation

On a regular basis, for example, quarterly, facilities should compare actual procedure volumes to procedure volumes reported in the appropriate facility account(s) for the Fluoroscopy DIR over a specific period of time. If total procedure volumes match and the Station Name of each connected fluoroscope is represented, it is likely that no further comparison is needed. If there is a mismatch, comparisons at the level of Station Name or even Study Description | Requested Procedure Description may be required for troubleshooting. The “Fluoro/DR Summary of Data Submitted” tool in the DIR Portal can be used to perform periodic data validation.

### 6.3 Facility process health monitoring

One other strategy participating facilities may consider to minimize interruptions in data flow from the facility to the DIR Central Server is to institute process health monitoring. An example of such a process is automated email alerts that notify the site administrator and diagnostic medical physicist if there is a failure to transfer data from PACS or an RDIM to the TRIAD® Site Server. The local facility informatics architecture will dictate what types of site process health monitoring can be implemented.

## 7 STEP 6. CLINICAL PROCEDURE NAME MAPPING

Facilities that participate in the Fluoroscopy DIR have their own internal facility clinical procedure “names” (a.k.a. procedure description, study description, etc.) for fluoroscopic procedures they perform. Clinical procedure mapping is an ongoing process that allows for procedures that are named differently at different sites to be analyzed and compared with other sites by mapping site‐specific facility clinical procedure names to a common lexicon, ACR Common in the case of the Fluoroscopy DIR. The Lead Fluoroscopy Technologist, often a senior fluoroscopy radiologic technologist, can help with procedure mapping, and the Physician Champion can provide support, as detailed knowledge of clinical fluoroscopy procedures is required to accurately map clinical procedures names.

All Fluoroscopy DIR participating sites are encouraged to perform mapping at the corporate account level. Clinical procedure name mapping performed at the corporate account level will apply to all facilities under the corporate account. This requires that any user who performs or maintains clinical procedure name mapping have the Service User profile assigned to their user account. Any mapping previously performed at the facility level will be overwritten if a Service User performs mapping at the corporate account level. Only users with a Corporate Account Administrator or Service User role are permitted to perform clinical procedure name mapping at the corporate account level.

Procedure mapping is executed in the Exam Name Mapping tool provided by the ACR within the DIR Portal. The Exam Name Mapping Tool User Guide, also found in the DIR Portal, is a useful reference for the mapping process. Using the Exam Name Mapping tool, there are two methods that can be used to map procedures.

### 7.1 Option 1. Map facility clinical procedure names using the Exam Name Mapping tool interface

With this method, facility clinical procedure names are mapped to the ACR Common lexicon within the Exam Name Mapping tool interface. Facility clinical procedure names are listed in the Exam Name Mapping tool as.

#### 7.1.1 Study description | requested procedure description

These are two separate fields in the DICOM metadata commonly associated with the facility clinical procedure name that may be populated differently depending on the clinical workflow of the facility. Either or both of these fields may contain the desired information. This can, however, increase the number of unique facility clinical procedure names that require mapping, as any unique combination of the two fields will generate another facility clinical procedure name to be mapped within the Exam Name Mapping tool. If a facility sends RDSRs to the TRIAD® Site Server via an RDIM, clinical procedure name mapping within the RDIM should be disabled, unless the most current version of ACR Common is used for mapping within the RDIM. Otherwise, clinical procedure name mapping within the RDIM may generate many more unique combinations of Study Description | Requested Procedure Description, which will substantially increase the number of facility clinical procedure names to be mapped within the Exam Name Mapping tool.

### 7.2 Option 2. Map clinical procedure names outside the exam name mapping tool interface

The Exam Name Mapping tool provides the ability to export all exam name mapping to Excel, and to download the ACR Common lexicon. Once downloaded, a facility can map clinical procedure names to the ACR Common lexicon in the downloaded spreadsheet, then upload the mapping back into the Exam Name Mapping tool. Many facilities find this more convenient than mapping each facility clinical procedure name individually in the Exam Name Mapping tool interface.

There are unique codes in the ACR Common lexicon that can be used to exclude specific facility clinical procedure names from future analysis. In the case of interventional fluoroscopy procedures, mapping a facility clinical procedure name to the code **9999998,'INV‐FLUOR. Unwanted’** will exclude that procedure from future analysis.

Facility clinical procedure name mapping will be very time intensive in the first few months after a site joins the Fluoroscopy DIR, and time requirements for mapping will decrease over time, although ongoing maintenance is required. Facility clinical procedure name mapping should be reviewed regularly, with more frequent reviews early on in the participation process.

If a facility makes sweeping global changes to facility clinical procedure names locally, it may be useful to request an internal mapping between old facility clinical procedure names and new facility clinical procedure names through your IT Specialist or a systems analyst, which can then be used to do a bulk update of facility clinical procedure name mapping in the Fluoroscopy DIR.

### 7.3 Accommodating varying levels of specificity in facility clinical procedure names

One of the major challenges for mapping of facility clinical procedure names during the pilot was the widely varying levels of specificity among the pilot sites, ranging from only about 25 different procedure names at one pilot site to over 100 different procedure names at two different pilot sites. For comparison, ACR Common currently contains 206 different interventional fluoroscopy procedures. The pilot team is currently working with the ACR to implement a “roll up” concept in which the ACR Common procedure names, the most specific level, all roll up to one or more categories that are more general. Sites that do not have sufficient specificity to map facility clinical procedure names to individual ACR Common procedures can map to one of the more general roll up categories. The current plan is to conduct data analysis and present summary statistics for both the ACR Common procedures (when at least 50 procedures comprising data from at least three sites have been received) and for the roll up categories.

## 8 STEP 7. DATA REPORTS AND ANALYSIS

Numerous data reports are available in the Summary of Data Submitted tab of the DIR portal. These reports provide graphical feedback and basic summary statistics at varying levels of detail, from individual Common procedure codes to “roll up” categories grouping similar procedures (e.g., all hepatic embolization). These reports will be very useful to diagnostic medical physicists in the performance of fluoroscopy dose audits^15^, ^16^ for the sites they support.

Currently, reports are provided on primary fluoroscopy dose indices, including reference air kerma (K_a,r_), air kerma area product (P_KA_), and fluoroscopy time; and for derived indices including:

$$K_{a,r}“\text{rate}”=K_{a,r}/\mathrm{fluroscopy}\;\mathrm{time};$$

$$\text{Average x - ray field size in the plane of the reference point}=P_{\text{KA}}/K_{a,r};$$

Percent of irradiation events using added filtration.

A suite of comparative benchmark reports similar to those available for the CT DIR will be provided to facilities as soon as a sufficient amount of fluoroscopic dose data from participating facilities becomes available.

## 9 CLOSING THOUGHTS

A successful launch of the Fluoroscopy DIR is a collaborative effort requiring a team with members that possess the requisite expertise in interventional radiology, diagnostic medical physics, and clinical radiology informatics. Participation in the Fluoroscopy DIR is a key aspect of a quality assurance program in fluoroscopy, as it allows sites to compare their practice to regional and national benchmarks, which we hope will ultimately result in a reduction in variability in radiation usage for procedures performed using fluoroscopy and drive the adoption of best practices for fluoroscopically guided procedures.

The qualified diagnostic medical physicist can be most effective in the implementation process if they understand both the physics aspects and the clinical aspects of fluoroscopically guided procedures. Devoting time to observing clinical fluoroscopy procedures is a very effective way for the diagnostic medical physicist to become more familiar with the clinical aspects of fluoroscopy.

## CONFLICT OF INTEREST

A. Kyle Jones, Ph.D. is President of FluoroSafety. FluoroSafety and its offerings are not discussed in the manuscript. Dr. Simanowith, Mr. Gress, and Ms. Zacharias‐Andrews are employees of the American College of Radiology. The other authors report no conflicts of interest or financial disclosures outside of their employment at their respective institutions.

## AUTHOR CONTRIBUTION

All listed authors have:

- Made substantial contributions to the conception or design of the work and the acquisition, analysis, and interpretation of data for the work; AND
- Drafted the work or revised it critically for important intellectual content;
- Approved the final version to be published; and
- Agreed to be accountable for all aspects of the work in ensuring that questions related to the accuracy or integrity of any part of the work are appropriately investigated and resolved.

## ABBREVIATIONS

ACR DIR central server, *A server maintained by the ACR that receives data transmitted from individual TRIAD Site Servers, analyzes the data, and hosts the database of processed DIR data*.

TRIAD® Site Server, *A physical or virtual server hosted by a participating site on which the TRIAD® software is installed*.

SCU, *Service Class User, in DICOM this is the device that uses a DICOM service, for example, for the image storage service, sends images*.

SCP, *Service Class Provider, in DICOM this is the device that provides a DICOM service, for example, for the image storage service, receives images*.

RDSR, *Radiation Dose Structured Report, a DICOM object that contains detailed, structured information describing each irradiation event during an X‐ray imaging procedure*.

HL7, *Health Level Seven, an international standards development organization that develops standards for interoperability between health information systems*.

RDIM, *Radiation Dose Index Monitoring system, a data system that receives, analyzes, and stores radiation dose information from medical imaging procedures*.

ACR Common, *A standard lexicon for medical imaging procedures, organized along multiple dimensions such as body part, modality, and organ*.

ACR National Radiology Data Registry (NRDR^TM^), *The data warehouse for all ACR Registries, including, but not limited to, the Dose Index Registry (DIR), Lung Cancer Screening Registry (LCSR), and the General Radiology Improvement Database (GRID). The NRDR^TM^ is a Qualified Clinical Data Registry (QCDR) for Merit‐based Incentive Payment System (MIPS) Reporting*.

AET, *Application Entity Title, a string of characters that uniquely identifies a DICOM host running a specific DICOM service*.

IP address, *Internet Protocol address, a series of numbers in the format xxx.xxx.xxx.xxx that uniquely identifies a device on a network*.

Station Name, *The host name of a medical imaging device, which is included in tag (0008,1010) of the General Equipment Module in the DICOM metadata of images created by the device. This tag is not mandatory, but is populated by many fluoroscopes*.

Study Description, *One of two DICOM metadata fields (tag 0008,1030) that may contain facility clinical procedure names. The local configuration of the imaging device may determine what information is stored in this field, if any. This tag is not mandatory, but is populated by many fluoroscopes*.

Requested Procedure Description, *One of two DICOM metadata fields (tag 0032,1060) that may contain facility clinical procedure names. Part of the Modality Performed Procedure Step (MPPS) modules. The Requested Procedure Description is often created and sent via Modality Worklist (MWL) by the Radiology Information System (RIS) or other hospital information system based on a dictionary of possible facility clinical procedures. This tag is not mandatory, but is almost always populated if a hospital information system is used to schedule procedures*.

DIR Portal, *Interface through which users interact with the Fluoroscopy DIR and the ACR DIR Central Server. Clinical facility procedure name mapping is done through the DIR Portal, and summary data reports can be found in the DIR Portal*.

Facility clinical procedure name, *The name assigned to a specific given clinical procedure by a site, for example, PERC NEPRHO PLC. Facility clinical procedure names may differ at different facilities within the same corporate site, and will almost certainly vary among different corporate sites*.

ACR Common procedure name, *The standard procedure name used in the ACR Common lexicon, all sites map their unique facility clinical procedure names to standard ACR Common procedure names*.

Device Observer Name, *One of the Device Observer Identifying Attributes in the Observer Context of the Projection X‐ray Radiation Dose Structured Report. Defaults to the Station Name from the General Equipment Module. This tag is not mandatory, but is populated by many fluoroscopes*.

Digital Imaging and Communications in Medicine (DICOM), *The international standard to transmit, store, retrieve, print, process, and display medical imaging information*.

Picture Archiving and Communication System (PACS), *A system consisting of hardware, software, and networking that allows the storage of medical images from multiple modalities and devices, viewing and manipulation of these images by users at multiple locations, and retrieval of images to other DICOM hosts*.

## Supporting information


**Data S1**. Checklists for use in preparing to launch the ACR Fluoroscopy DIR at your site.
