# Supplementary material for: A diagnostic medical physicist’s guide to the American College of Radiology Fluoroscopy Dose Index Registry
Source: J Appl Clin Med Phys. 2021 Mar 25;22(4):8–14. doi: 10.1002/acm2.13227 (PMC8035558; doi:10.1002/acm2.13227)
Supplement: Supplementary file 1 — Data S1. Checklists for use in preparing to launch the ACR Fluoroscopy DIR at your site. [file ACM2-22-8-s001.docx]

Readiness checklist for participation in the ACR Fluoroscopy DIR

|  | Decision made on networking configuration to route RDSR to TRIAD® Site Server |
| --- | --- |
|  | List of fluoroscopes to connect to Fluoroscopy DIR assembled |
|  | Identify physical or virtual server to run the TRIAD® Site Server software |
|  | Physician Champion identified |
|  | Diagnostic Medical Physicist identified |
|  | IT Specialist identified |
|  | TRIAD® Site Server Administrator identified (may be the same as above) |
|  | Lead Fluoroscopy Technologist identified |

Fluoroscope configuration checklist

|  | Fluoroscope configured to produce RDSR |
| --- | --- |
|  | Fluoroscope configured to transfer RDSR to appropriate network destination(s) |
|  | Fluoroscope listed in the TRIAD® Site Server configuration |
|  | Fluoroscope and workflow configured to include the appropriate facility clinical procedure name in either the Study Description or Requested Procedure Description |
